# Mechanical nociceptive assessment of the equine hoof after navicular bursa anesthetic infiltration validated by bursography

**DOI:** 10.1371/journal.pone.0269532

**Published:** 2022-06-07

**Authors:** Letícia de Oliveira Cota, Bruno Dondoni Malacarne, Lucas Antunes Dias, Antônio Catunda Pinho Neto, Maria Luiza Arruda Kneipp, Marina Alcântara Cavalcante, Marina de Souza Luz da Cunha, Cahuê Francisco Rosa Paz, Armando de Mattos Carvalho, Rafael Resende Faleiros, Andressa Batista da Silveira Xavier

**Affiliations:** 1 Department Veterinary Clinic and Surgery, EQUINOVA Research Group, Veterinary School, Universidade Federal de Minas Gerais, Belo Horizonte, Minas Gerais, Brazil; 2 National Council for Scientific and Technological Development - CNPq, Brasília, Distrito Federal, Brazil; Universidade do Porto Instituto de Biologia Molecular e Celular, PORTUGAL

## Abstract

The analgesic specificity of navicular bursa (NB) anesthetic infiltration is still questionable. The study aimed to determine the mechanical nociceptive threshold of non-specific analgesia in the dorsal lamellar stratum, as well as in the sole, coronary band, and heel bulbs of the hoof, after navicular bursa anesthetic infiltration. Six healthy horses with no clinical or radiographic changes of the digits and no communication between the NB and the distal interphalangeal joint, were used. After random selection, the NB of one of the forelimbs was infiltrated with 2% lidocaine and the contralateral one with lactated ringer’s solution. Contrast was added to confirm radiographic infiltration. The mechanical nociceptive threshold was determined using a portable pressure dynamometer, before and at various times after the infiltration, in 10 points of the hoof. The effects of time and treatment were verified by ANOVA (P<0.05). There was no statistical difference in the values of the mechanical nociceptive threshold (P>0.05) in all regions evaluated. However, in one of the six hooves that receives lidocaine, complete absence of response to the painful stimulus (maximum force of 6 Kg over an area of 38.46 mm^2^, for a maximum of 4 seconds) was observed in the dorsal lamellae between 30 and 60 min after infiltration. In conclusion, lidocaine infiltration of NB did not promote significant increases in the nociceptive threshold of the sole, coronary band, bulbs of the heel and dorsal lamellae clinically healthy horses. However, the occurrence of analgesia in one of the six hooves subjected to NB anesthesia indicates that the technique may not be fully specific in few horses.

## Introduction

Even with advances in diagnostic imaging that accurately locate lesions in the distal portion of the equine digit, determining the main source of pain is still challenging. Diagnostic analgesia is considered fundamental to precision in identifying these lesions [1–3].

The specificity of diagnostic blocks of the distal portion of the equine digit is still debatable, as is the extent of the desensitized regions [2]. Perineural anesthesia of the digital palmar nerve (DPN) at the height of the alar cartilage was considered to desensitize only the third or palmar half of the hoof [4]. However, later studies found that the entire sole [5], distal interphalangeal joint (DIJ) [6], and dorsal lamellar layer [7] were also anesthetized. Moreover, DIJ intrasynovial anesthesia may also promote analgesia in other structures of hoof like the podotroclear apparatus [8–10], the hoof sole [5,11], the distal portion of the deep digital flexor tendon (DDFT) [12], and the dorsal lamellar stratum of the hoof [13]. The diagnostic analgesia of the navicular bursa (NB) causes desensitization of the synovial cavity and podotroclear apparatus [9,12]. However, nonspecific desensitization of the sole [14,15], of the DDFT in the insertion of the distal phalanx [12], and even the DDFT into the NB may occur after NB analgesia [16,17]. As such, there remain doubts regarding the non-specificity of anesthetic infiltration of NB in other regions of the hoof.

Of the regions that may suffer from non-specific analgesia after perineural and intrasynovial infiltrations in the hoof, the dorsal lamella region stands out. This is due to the increasing occurrence of endocrinopathic laminitis cases of slow evolution, which may, in the initial stages, present lameness without characteristic radiographic signs that allow a definitive diagnosis of this condition [18]. Recent studies by our group demonstrated that the lamella regions of healthy horses can be desensitized after palmar digital nerve perineural anesthesia [7], and that some horses may show signs of lamellar analgesia after anesthetic infiltration of the distal interphalangeal joint [13].

The present study hypothesized that the anesthetic infiltration of NB, without extravasation of the drug, does not promote non-specific analgesia in other areas of the hoof, such as the dorsal lamella regions and sole. Therefore, the objectives of the present study were to: use pressure algometry to evaluate the mechanical nociceptive threshold (MNT) and determine whether the anesthetic infiltration of NB with 2% lidocaine hydrochloride can increase the MNT of the dorsal lamella region of the hoof, as well as the sole, bulbs, and coronary band.

## Materials and methods

### Animals and experimental design

The experiment was approved by the Ethics Committee on Animal Use of Universidade Federal de Minas Gerais (UFMG) (Approval No.360, February 27, 2018). Six mixed-breed untrained and unexercised horses of varying sex and neuter status (4 mares, 1 gelding, and 1 stallion), owned by the Veterinary School of UFMG, were used in randomized blocks and repeated measurements over time. The horses were 7–13 years of age (median, 9 years), 142–164 cm tall (median, 146 cm), and weighed 350–460 kg (median, 355 kg). All horses were used to basic handling including hoof trimming.

All horses were considered clinically healthy, as determined via physical and orthopedic examinations. A complete lameness examination was performed on all the horses to ensure that no lameness was present [19].

The anesthetic infiltration effects of NB in the hoof were compared using a cross-over experimental design. Treatments were randomized in each horse. One forelimb NB received 2 mL of 2% lidocaine (Xylestesin 2% ^®^—Laboratório Cristália) and 1 mL of 300 mg iodine/mL contrast solution (Omnipaque^®^—GE Realthcare). The NB of the contralateral limb received 2 mL of lactated Ringer’s solution and 1 mL of contrast. Contrast solution was used for radiographic confirmation of accurate access to the NB. Communication between the synovial structures was considered when extravasation of contrast was found into the DIJ or flexor tendon sheath. In that case, the horse was excluded from the experimentation. The veterinarian responsible for administering the infiltrations was blinded from the grouping.

### Hoof preparations

Before the study, horses were restrained in stocks, and their hooves were cleaned and trimmed for geometric balance. Hoof preparation for pressure algometry testing was performed as previously described by Paz et al. [7]. Horses were sedated with detomidine (20 μg kg−1 intravenous (IV); Detomidin, Sintec), followed by intramuscular (IM) administration of tetanus antitoxin (5000 IU; Lema Injex), and aseptic preparation of the hoof capsule. Two hoof wall defects were made in the center of the dorsal hoof wall of each forefoot, using a 10-mm drill bit mounted onto a drill (Dewalt 12v max). The first defect was located 2 cm (L2) distal to the coronary band and the second 4 cm (L4) distal to the coronary band. Hoof wall defects penetrated only the insensitive horn, stopping immediately before reaching the sensitive lamellae, which was visually identified as a soft tissue. Using a hoof knife, the sole of each hoof was prepared by removing the keratinized layer until the sensitive tissue was reached near the solar corium, at three different points close to the palmarolateral, palmaromedial, and dorsal borders of the sole. The pressure algometry was used over these defects to determine that it has reached the same MNT obtained on the bulbs and coronary band of the respective hoof. If the MNT of the defect was larger to the bulbs or coronary band, careful penetration was continued for approximately to 1-2mm deeper.

### Anesthetic infiltration of the navicular bursa

Twenty-four hours after creating the defects the animals were restrained in stocks without sedation. Since local anesthesia of the skin above the coronary band was not performed to avoid alteration of the MNT on the bulbs, nose and skin twitch were used, and the eyes were covered for needle placement. After aseptic preparation of the hoof, the limb was raised and maintained elevated during the entire procedure. One assistant held the limb with care without letting the horse move the limb. If the horse reacted to the needle placement, attempts to keep the limb in flexion was made until the infiltration was completed. If the horse pulled the limb, attempts were made to catch it again and the procedure was continued when appropriated restrain was obtained. On average, the duration of bursocentesis was 3 minutes.

**Fig 1:**
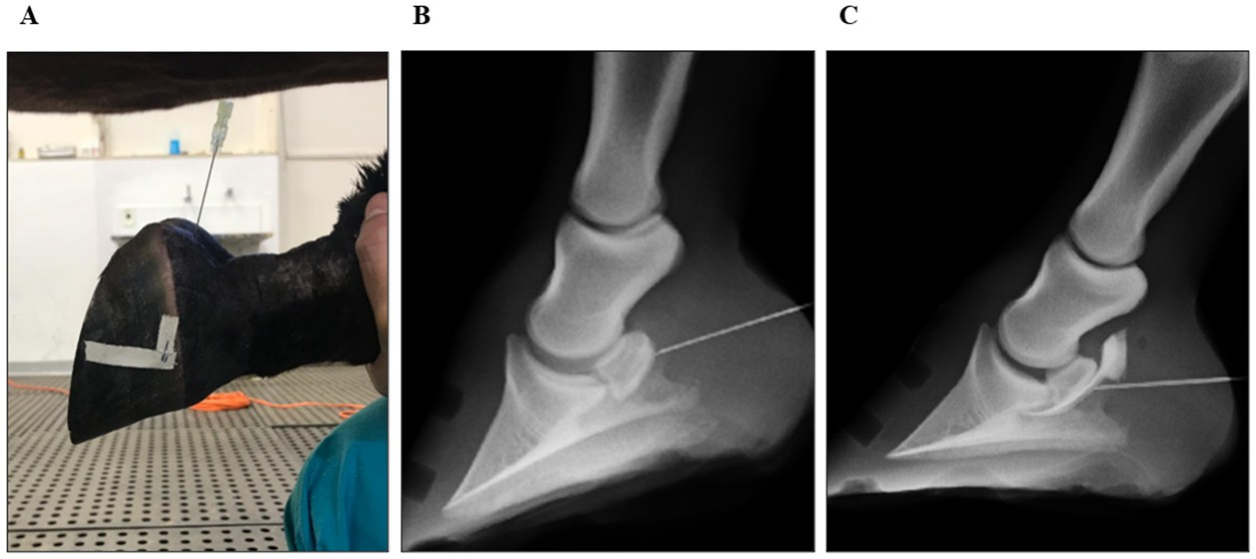
Equine forelimb bursography images. **(A)** Lateral aspect of bursocentesis performed as described by Verschooten et al. [20]. A 20G 3½ spinal needle, inserted in the sagittal plane proximal to the coronary edge of the bulb, crossing the skin, digital cushion, and deep digital flexor tendon, towards the proximal third of the navicular bone, until it is significant. **(B)** Latero-medial radiography of the hoof during bursocentesis of the left forelimb, confirming the position of the spinal needle on the palmar-proximal surface of the navicular bone. **(C)** Navicular bursa bursography. Bursocentesis confirmation without leakage and without communication with the distal interphalangeal joint. It guarantees the reliability of the measurements of the mechanical nociceptive threshold.

Access to NB was performed as described by Verschooten et al. [20], using a 20G 3½ spinal needle inserted into the sagittal plane proximal to the coronary edge of the bulb, crossing the skin, digital cushion, and DDFT, and toward the proximal third of the navicular bone, until there was significant resistance (Fig 1A). Lateromedial radiography of the hoof was performed (EcoRay Co., Ltd 1060HF, Korea) to direct the needle to the center of the flexor surface of the navicular bone and avoid crossing the DDFT several times. NB infiltration was performed after radiographic confirmation of the needle apposition with the flexor surface of the navicular bone (Fig 1B). The infiltration was considered effective after complete NB marking in the immediate bursography, without extravasation of contrast to the external surroundings of the bursa (Fig 1C). Extravasation of contrast was evaluated immediately after the NB infiltration and 10 minutes later. The contrast started to lose definition and for this reason continued radiographic evaluation was not performed.

### Algometry

Pressure algometry was utilized to evaluate the MNT for each hoof. A portable dynamometer (Instrutemp 20kgf ITFG-5020), calibrated by the manufacturer for compression, was used to determine the force (kg) necessary to incite a response, as described for humans [21] and equines [7,13,22,23]. Using both hands, the dynamometer was applied with a constant increase in pressure at a 90° angle in relation to the contact surface. Pressure algometry was performed on each hoof at 10 different sites: coronary band (medial, lateral, dorsal regions), heel bulbs (medial and lateral), sole (dorsal, palmaromedial, and palmarolateral regions), and dorsal lamellae (2 cm and 4 cm distal to the coronary band). All limbs were loaded during assessments, except the soles. A 7-mm diameter flat metal tip was used for testing the coronary band and heel bulbs. A metallic cone tip (7mm base diameter) was used to evaluate the dorsal lamellae and sole regions [7].

To avoid environmental distractions and visual perception of the algometer being applied, a blindfold was applied over the eyes of each horse during each evaluation. The basal MNT for each foot was determined prior to the NB administration of each treatment. The minimum force required to stimulate the withdrawal of the limb was recorded. Sites were assessed at 13 pre-selected times, beginning 10 min pre-injection (baseline), followed by 5, 10, 15, 20, 30, 60, 90, 120, 150, 180, 210, and 240 min post-injection. To avoid tissue injury and trauma to the foot of the horse, a maximum force of 6 kg was set to determine responsiveness. This cutoff value was based on previous studies that used algometry [7,22].

Preparation of the injectate was performed by an individual not blinded to the treatments. NB injections and dynamometer measurements were also performed by a single investigator blinded to the randomized treatments. Moreover, two additional evaluators, also blinded to the treatments, recorded the minimal force applied to the hoof that caused limb withdrawal. The response was determined twice at each time interval of 2–4 s, and the average of the two evaluation was considered. When divergence in reading occurred between the 2 evaluators, the value was disregarded, and the procedure was repeated. If the difference between the values obtained in two evaluations was greater than 0,05 kg of force, a third measurement was performed. After the experimental period, all horses received phenylbutazone (4.4 mg kg−1, Ourofino) IV daily for 5 days.

### Statistical methods

The mean values of the MNT were compared using a specific software (Sigma Stat). ANOVA analysis of variance with repeated measurements of two factors was used to assess the difference between animals, times, groups, and between times and groups. However, in the absence of normal data distribution in the Shapiro-Wilk test, the effects of time in each group were analyzed using the Friedman test. Moreover, the effects of time in each group were compared separately using the Wilcoxon test (GraphPad Prism 7). For all analyses, <0.05 was considered significant.

## Results

NB infiltration was successfully performed in all six horses without sedation. Two mares were excluded from the experiment during the bursography. One mare has presented direct communication between the NB and the DIJ and the other a leak in the lateral portion of the proximal recess of the NB.

The mean baseline values (± standard deviation) of the MNT for each group for each area assessed are shown in Table 1.

**Table 1:** Mean baseline values (± standard deviation) of the mechanical nociceptive threshold (in kg) of the dorsal lamella (2 and 4 cm distal to the coronary band), sole, bulbs of the heel and coronary band of the forelimb hooves of healthy horses subjected to navicular bursa infiltration with lidocaine 2% or Ringer’s lactated solution.

	Lactated ringer		2% lidocaine	
	Mean	SD	Mean	SD
Heel bulbs lateral	0,50	0,14	0,40	0,17
Coronary band lateral	0,48	0,21	0,31	0,15
Coronary band dorsal	0,58	0,08	0,38	0,19
Coronary band medial	0,52	0,21	0,45	0,11
Heel bulbs medial	0,55	0,16	0,37	0,24
Dorsal lamellae 2 cm	0,37	0,18	0,37	0,19
Dorsal lamellae 4 cm	0,28	0,12	0,44	0,26
Sole dorsal	0,56	0,20	0,41	0,13
Sole medial	0,58	0,24	0,45	0,16
Sole lateral	0,59	0,27	0,50	0,19

There was no statistical difference (P>0.05) when comparing MNT of the control versus treatment group, between groups and times. The Wilcoxon test showed a difference between groups at baseline for the lateral coronary band (P = 0.03). However, this was not clinically significant, since there was no difference in the following measurement times after anesthetic infiltration of the NB.

**Fig 2:**
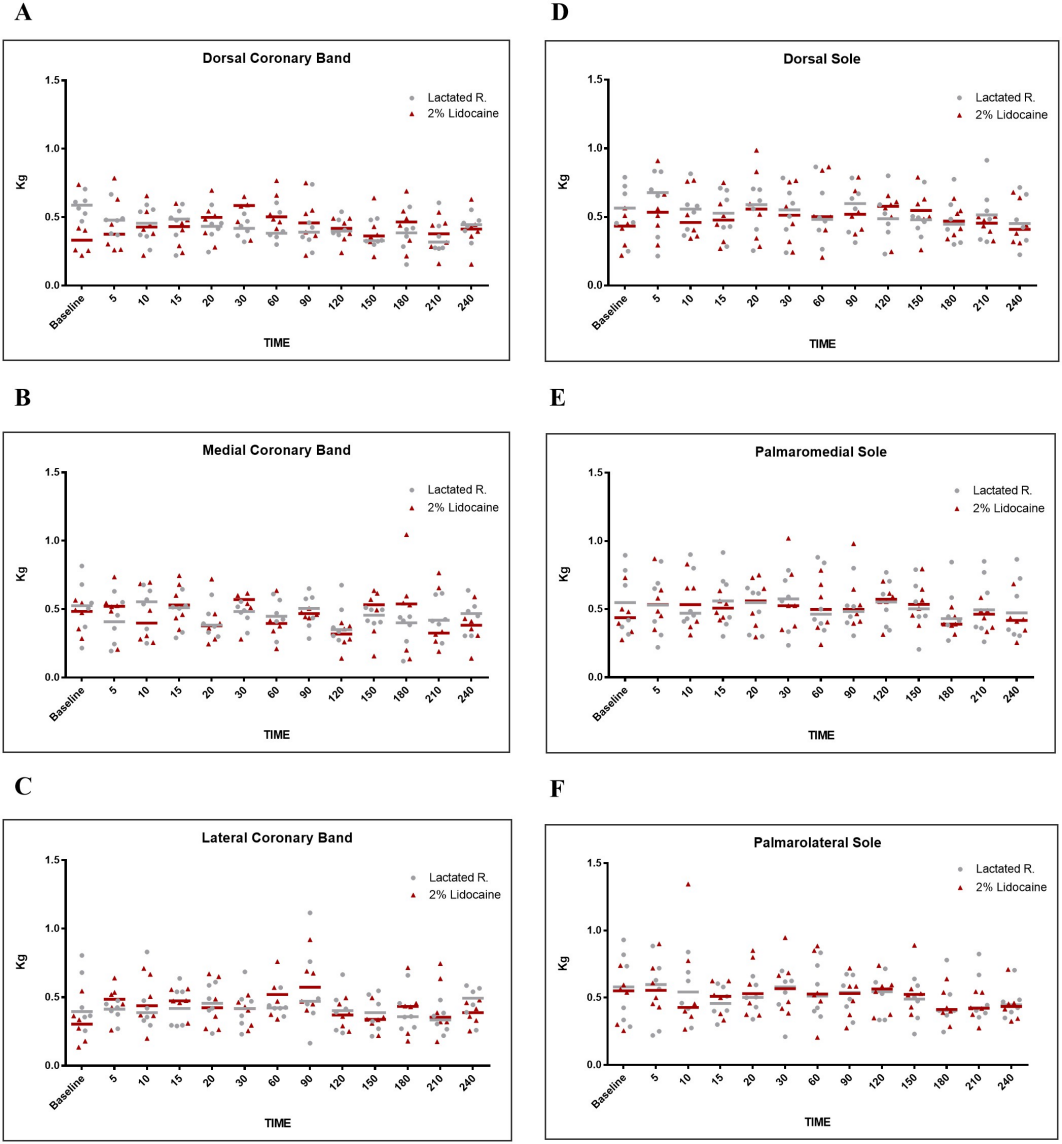
Graphical representation of mechanical nociceptive threshold (MNT) values, with mean values and median in kilograms (kg). Evaluation of coronary band: **(A)** dorsal, **(B)** medial, **(C)** lateral. Evaluation of sole borders: **(D)** dorsal, **(E)** palmaromedial, **(F)** palmarolateral, in healthy equine forelimbs, injected into navicular bursa with lactated Ringer’s solution or 2% lidocaine.

**Fig 3:**
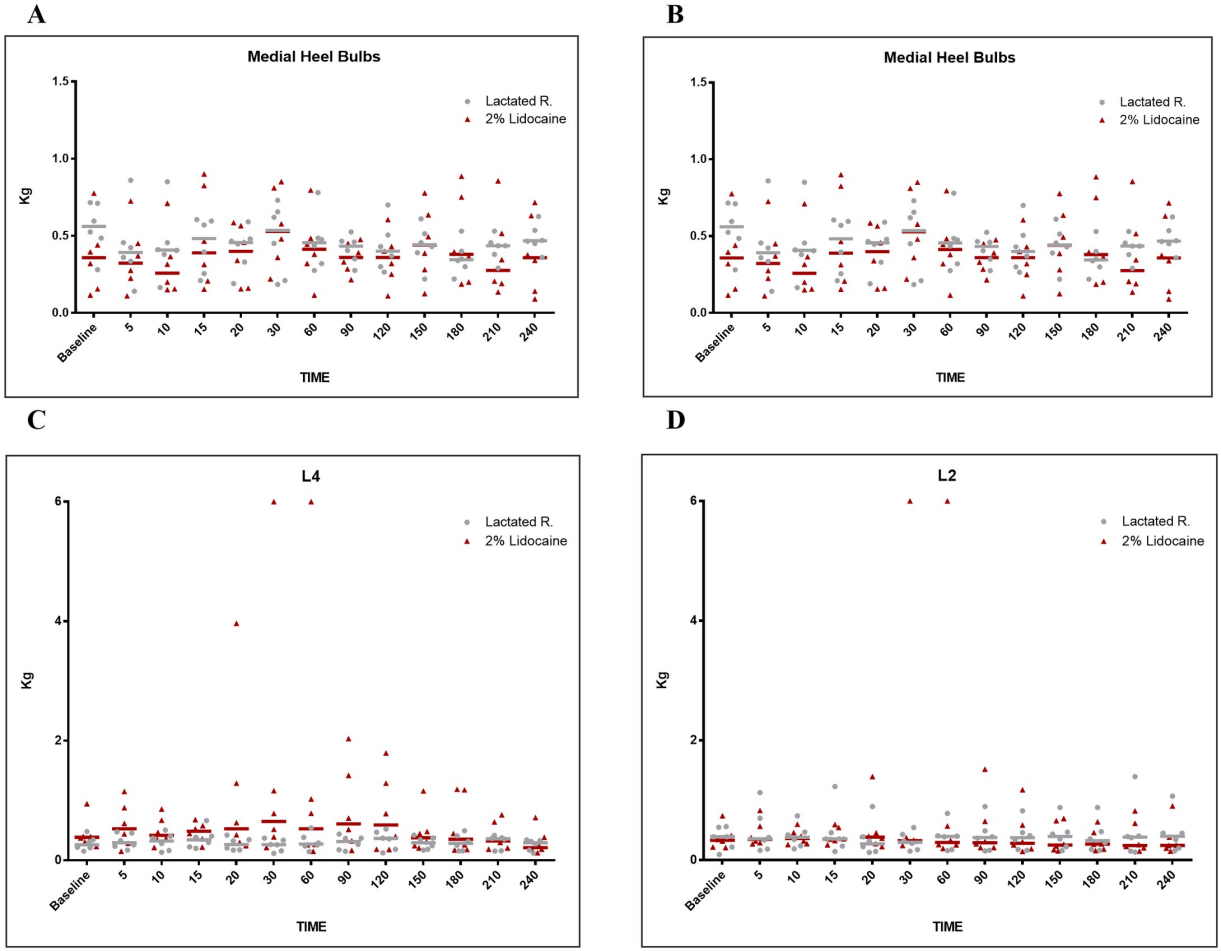
Graphical representation of mechanical nociceptive threshold (MNT) values, with mean values and median in kilograms (kg). Evaluation of heel bulbs: **(A)** medial, **(B)** lateral. Evaluation of dorsal lamellar regions (L): **(C)** L2 cm, and **(D)** L 4 cm, distal to the coronary band of hooves in healthy equine forelimbs, injected into navicular bursa with lactated Ringer’s solution or 2% lidocaine.

Through graphical analyses (Figs 2 and 3), it is possible to observe the individual values and median of the MNT of each group at each time. There was a slight fluctuation in the median over time in both groups. Most remained close to 0.5 kg in all regions while some rare individual measurements exceeded 1 kg of force. However, in the points of the dorsal lamellar layer, specifically in animal 3, a force of 3.96 kg was observed at L4, 20 min after anesthetic infiltration (Fig 3D), and a maximum value of 6 kg in L2 and L4 after 30 and 60 min (Fig 3C and 3D).

## Discussion

The study demonstrates that the anesthetic infiltration of the NB did not promote a significant increase in the MNT in the dorsal lamellar layer, sole, coronary band, and in the hoof bulbs in healthy horses under the studied conditions, corroborating the hypothesis studied. The individual temporal analysis of the MNT of each region evaluated in each animal allowed us to verify the maintenance of hoof sensitivity. Graphically, there are small variations and alternations in the median position of the MNT in the control group, as observed in the study by Paz et al. [7] and Malacarne et al. [13].

However, in the group of horses with anesthetized bursae, specifically in animal 3, signs of dorsal hoof wall analgesia were detected. Twenty minutes after anesthetic infiltration, there was a 9.9-fold increase in the MNT at point L4 compared to the baseline measurement. In this same animal, the maximum value of 6 kg was reached in the measurements of the dorsal lamellar tissue at points L2 and L4 performed at 30 and 60 min. Such findings, although statistically negligible, should be carefully analyzed from a clinical point of view, as they indicate that the technique used for infiltration of NB, even without anesthetic extravasation, may promote lamellar analgesia in some horses.

It is known that anesthetic diffusion occurs to adjacent tissues over time after drug infiltration of synovial cavities [24–26], which, supposedly, could explain the desensitization of the dorsal lamellar stratum of the hoof between 20 and 60 min after anesthetic infiltration of the NB in 1 of 6 animals evaluated (16.6%). According to Sack [27], the lamellar corium is innervated exclusively by the DPN without the participation of its dorsal branches. The DPN is further divided into superficial and deep branches, with the superficial branches innervating the lamellar corium of the quarters, bars, and heel. The deep branches would be responsible exclusively for the inversion of the dorsal lamellar corium through the 8th and 5th branches of the medial and lateral digital nerves, respectively. Both branches are located closely juxtaposed to the distal recess of the NB and impar ligament, and both follow the digital arteries towards the terminal arch through the solear foramen. Therefore, it would be plausible to think that an overflow of anesthetic in this region of the bursa could produce analgesia of the lamellar corium, especially in its dorsal region.

NB anesthesia non-specificity has been previously documented. In horses with screw-induced lameness, different degrees of hoof dorsal analgesia were observed after 10 min of anesthetic infiltration of the NB using mepivacaine and lidocaine [14,15]. A greater anesthetic volume (3.5 mL) contributed to the movement and pressures, by stretching the NB during dynamic examination, which may have favored the diffusion of the anesthetic abaxially. Schumacher et al. [14] also found that anesthetic volume had an important influence on the analgesic effects of anesthetic infiltration of the DIJ on dorsal sole pain, with greater desensitization when a greater volume of anesthetic solution was used. This greater diffusion may reach the additional branches of the deep DPN which innervates the dorsal sole region, and this may explain the differences between the models mentioned and the current one. The chosen anesthetic agent also influences the analgesic response. Hoerdemann et al. [28] demonstrated that despite skin desensitization occurring after perineural blockade with lidocaine, this did not fully resolve lameness in all horses, in contrast to mepivacaine. In the present study, lidocaine was used since mepivacaine access is limited in Brazil.

Of the present study, in combination with Schumacher et al. [11] and Sardari et al. [15] allows us to verify that non-specificities in the analgesic response can occur at 10 and 20 min according to the volume of solution administered after the NB anesthetic infiltration. Furthermore, clinical studies with magnetic resonance imaging and scintigraphy, suggest that NB diagnostic analgesia is effective in obtaining specific improvement in pain from the podotrochlear apparatus in all animals tested when evaluated in the first 5 min after anesthetic infiltration [12,16,29].

The technique used in this study for NB anesthetic infiltration was described by Verschooten et al. [20] as easy to perform and allowed an inexperienced veterinarian to obtain good success rates [5,14,30]. However, in this study, the technique used was elaborated. The importance of a synchronized team for execution must be emphasized. These observations are applicable mainly when performing NB blocks. The need to keep the horse alert to measure the MNT prevented the use of chemical restraint and/or local block. Despite proper use of physical restraint, it was difficult to keep the limb elevated and non-reactive during bursocentesis. There was also a deviation of the needle, especially when crossing the DDFT. Furthermore, there was also the applicator’s difficulty in verifying the angle with the hoof. In agreement with Kent Allen et al. [31], we observed that even when using anatomical references, radiography is still essential for guiding and repositioning the needle; and after obtaining resistance, for confirming apposition with the palmar surface of the navicular, before injection. Other recent techniques to access the NB were not addressed [32,33], since the focus of the study was to verify the effect of local anesthetic in the bursa independently of the assessment.

To validate the results, bursography is necessary. In the present study, one of the evaluations was invalid and had to be repeated because in one of the tested members, a contrast solution with lidocaine was deposited outside the NB on the palmar face of the DDFT. This promoted almost immediate desensitization of the sole and the dorsal lamellar layer, like what was observed in the study by Paz et al. [7] who performed DPN block.

In addition, two horses were removed from this experiment because they presented anatomical particularities of the bursa that could potentially interfere with the study results. One of them showed direct communication between the NB and DIJ, and the other showed leakage in the lateral portion of the proximal recess of NB. Kent Allen et al. [31] observed 11% and 18% of these changes in NB, respectively. Hontoir et al. [34], however, observed synovial communication between the DIJ and NB in only 5.3% of the evaluated members. In the study, radiographic examinations of these animals revealed communication and leakage in the proximal recess of the NB through the T-ligament. Although histologically it is not a ligament, the T-ligament directly connects the dorsal side of the DDFT and the palmar aspect of the middle phalanx, and its fibers reach the connection of the lateral and medial collateral sesamoidean ligaments that runs on the proximal border of the navicular bone and intercepts the digital flexor tendon sheath, DIJ and NB [34]. Supposedly, the communication of the DIJ and NB, or even an overflow of NB, could occur through the T-ligament due to its indirect connection to the proximal edge of the navicular bone. Although uncommon, in these cases, the infiltrated solution is not restricted to NB and generates non-specificities in the test. In general, the use of bursography in the anesthetic and drug infiltrations of NB is emphasized so that false interpretations and failures, or low responses to treatment, do not occur.

Among the forms of analysis and quantification of nociception in horses, pressure algometry is the method best suited to evaluating conscious animals [35]. The baseline values of the regions evaluated in the hoof of the animals in this study were similar to those reported by Paz et al. [7] and Malacarne et al. [13] in crossbred animals. Therefore, the pressure algometry methodology showed repeatability and effectiveness in assessing the anesthetic effect without causing tissue damage.

The present study’s methodological limitations should be highlighted. After NB infiltration, the horses were not actively moved, so the distended NB did not suffer the pressure that it would normally receive in dynamic examination, which may interfere with the diffusion. Healthy horses were used in the present study. Just as there are individual variations, it is not known whether horses with injuries have anatomical and nociceptive changes that may modify the sensitivity and, consequently, the result of NB anesthetic infiltration, or change the diffusion of solutions. Also, a pharmacological distribution between DIJ and NB has already been observed to be inversely proportional to the severity of radiographic lesions [26], indicating that more advanced lesions reduce diffusion. Besides, this is an initial study with a limited number of animals, requiring the use of non-parametric statistics that are known to have less power when compared to tests for normally distributed data. Thus, new controlled studies with larger sample sizes for the dynamic examination of horses after blocks, or even clinical studies in horses with lesions in the podotrochlear apparatus, are still necessary to further the understanding of this diagnostic analgesia technique’s specificity.

## Conclusions

There was no significant increase in the MNT of the coronary band, heel bulbs, sole, or the dorsal lamellae after NB anesthetic infiltration of healthy horses. However, signs of analgesia were observed in the dorsal lamellar stratum of the hoof in a horse after twenty minutes of NB anesthetic infiltration with lidocaine. This result, although preliminary, indicates caution in the interpretation of the diagnostic analgesia technique 15 min after the infiltration of the NB.

## Supporting information

S1 Table Mechanical nociceptive threshold (in kg) of the dorsal lamella (2 and 4 cm distal to the coronary band), sole, bulbs of the heel and coronary band of the forelimb hooves of healthy horses subjected to navicular bursa infiltration with 2 ml of lidocaine 2% or Ringer’s lactated solution. (XLSX)

S2 Table (PDF)
